# *Schistosoma mansoni* FES Tyrosine Kinase Involvement in the Mammalian Schistosomiasis Outcome and Miracidia Infection Capability in *Biomphalaria glabrata*

**DOI:** 10.3389/fmicb.2020.00963

**Published:** 2020-06-11

**Authors:** Naiara Clemente Tavares, Sandra Grossi Gava, Gabriella Parreiras Torres, Clara Ênia Soares de Paiva, Bernardo Pereira Moreira, Felipe Miguel Nery Lunkes, Langia Colli Montresor, Roberta Lima Caldeira, Marina Moraes Mourão

**Affiliations:** ^1^Grupo de Helmintologia e Malacologia Médica, Instituto René Rachou, Fundação Oswaldo Cruz, Belo Horizonte, Brazil; ^2^Moluscário Lobato Paraense, Instituto René Rachou, Fundação Oswaldo Cruz, Belo Horizonte, Brazil

**Keywords:** *Schistosoma mansoni*, feline sarcoma, kinase, signaling pathways, development, oogenesis, RNA interference, granuloma

## Abstract

Schistosomiasis is a neglected tropical disease (NTD) caused by helminthes from the *Schistosoma* genus. This NTD can cause systemic symptoms induced by the deposition of parasite eggs in the host liver, promoting severe complications. Functional studies to increase knowledge about parasite biology are required for the identification of new drug targets, because the treatment is solely based on praziquantel administration, a drug in which the mechanism of action is still unknown. Protein kinases are important for cellular adaptation and maintenance of many organisms homeostasis and, thus, are considered good drug targets for many pathologies. Accordingly, those proteins are also important for *Schistosoma mansoni*, as the parasite relies on specific environmental signals to develop into its different stages. However, the specific roles of protein kinases in *S. mansoni* biology are not well understood. This work aims at investigating the tyrosine-protein kinase FES (Feline Sarcoma) functions in the maintenance of *S. mansoni* life cycle, especially in the establishment of mammalian and invertebrate hosts’ infection. In this regard, the verification of Sm*fes* expression among *S. mansoni* stages showed that Sm*fes* is more expressed in infective free-living stages: miracidia and cercariae. Schistosomula exposed to SmFES-dsRNA *in vitro* presented a reduction in movement and size and increased mortality. Mice infected with Sm*fes*-knocked-down schistosomula exhibited a striking reduction in the area of liver granuloma and an increased rate of immature eggs in the intestine. Female adult worms recovered from mice presented a reduced size and changes in the ovary and vitellarium; and males exhibited damage in the gynecophoral canal. Subsequently, miracidia hatched from eggs exposed to SmFES-dsRNA presented changes in its capability to infect and to sense the snail mucus. In addition, the SmFES RNAi effect was stable from miracidia to cercariae. The establishment of infection with those cercariae reproduced the same alterations observed for the knocked-down schistosomula infection. Our findings show that SmFES tyrosine kinase (1) is important in schistosomula development and survival; (2) has a role in adult worms pairing and, consequently, female maturation; (3) might be essential for egg antigen expression, thus responsible for inducing granuloma formation and immunomodulation; and (4) is essential for miracidia infection capability. In addition, this is the first time that a gene is kept knocked down during three different *S. mansoni* life stages and that a tyrosine kinase is implicated in the parasite reproduction and infection establishment in the mammalian host. Accordingly, SmFES should be explored as an alternative to support schistosomiasis treatment and morbidity control.

## Introduction

Schistosomiasis is a neglected tropical disease (NTD) caused by a trematode from the *Schistosoma* (Platyhelminthes: Trematoda) genus. This NTD is reported in 78 countries, and it is estimated that 700 million people live in endemic areas (World Health Organization, 2018).

This disease can cause disabling systemic morbidity resulting in disability-adjusted life year (DALY) loss ranging from 1.7 to 4.5 million (Steinmann et al., 2006; Hotez et al., 2014). Schistosomiasis morbidity is induced by granuloma formation in response to parasite eggs trapped in the host tissues (Burke et al., 2009). The initial inflammation can progress to fibrosis causing injuries and additional complications that can lead to anemia, malnutrition, impaired childhood, damage in cognitive function, and great loss of quality of life, thus causing a significant socioeconomic impact (King and Dangerfield-Cha, 2008; Burke et al., 2009).

Currently, schistosomiasis treatment is performed using praziquantel (PZQ). PZQ administration is low cost and efficient and presents minor side effects. However, PZQ efficacy is dependent on the stage of the parasite, mating status, and sex of the adult worms. Specifically, less effect is observed in eggs and immature worms (Watson, 2009; Thétiot-Laurent et al., 2013). The low PZQ efficacy in immature worms is considered a critical disadvantage because the immature worms develop into adults, causing future morbidity, which may have severe impacts on the disease eradication in endemic areas (Colley et al., 2014). Accordingly, studies for elucidating parasite biology and searching for new therapeutic strategies are needed (Engels et al., 2002; Caffrey and Secor, 2011).

Protein kinases (PKs) are drug targets for human diseases such as cancer and are also studied as promising targets to combat parasitic diseases (Bahia et al., 2006; Boyle and Koleske, 2007; Beckmann et al., 2012). Some intracellular proteins such as glutathione-*S*-transferase, antioxidative thioredoxin peroxidases, and manganese superoxide dismutase are essential to promote the homeostasis and cellular adaptations in *Schistosoma* to environmental signals (Liu et al., 2006; Han et al., 2009). Hence, PKs are considered essential owing to their central roles in mediating those intracellular responses (Wilkinson and Millar, 2000; Hotez et al., 2014). In *Schistosoma mansoni* Sambon, 1907, eukaryotic PKs (ePKs) correspond to 1.9% of predicted parasite proteome, and only 14.9% of them have experimental evidences (Grevelding et al., 2018). To date, studies have demonstrated that PKs participate in host sensing and invasion, oxidative stress response, tegument maintenance, development, and reproduction of *S. mansoni* (Andrade et al., 2014; Ressurreição et al., 2014, 2015; Avelar et al., 2019).

Tyrosine kinases (TKs) are known as proteins that play a role in cell division regulation, differentiation, metabolism, and development of many organisms (Levitzki and Gazit, 1995). Among *S. mansoni* kinome, 34 PKs (7.1%) are from the TK group (Andrade et al., 2011) and are described as essential for parasite growth, development, and survival (Zwick et al., 2001; Bahia et al., 2006; Katz et al., 2013). TKs are classified as receptor (RTK) or cytoplasmatic (CTK) (Dissous et al., 2007). CTK comprises 11 families; among them, only SmFES (Smp_332370) is identified as a member of the family FES/FPS/Fer [feline sarcoma (FES), Fujinami poultry sarcoma (FPS), and FES related (Fer)] (Andrade et al., 2011). The FES/FPS/Fer family is known to be involved in cell interactions and cytoskeleton rearrangement (Greer, 2002). Also, it is described that Sm*fes* is expressed in the miracidium terebratorium and schistosomulum and cercaria tegument (Bahia et al., 2007). However, its function in the establishment of mammalian/invertebrate infection and parasite development remains unknown.

The characterization of mechanisms and molecules involved in processes that regulate the parasite’s cellular functions and host–parasite interaction are essential for the understanding of *S. mansoni* biology. As PKs are considered promising drug targets and studies demonstrate that some PKs are related to *S. mansoni* development and survival, this work aims at characterizing the TK SmFES to determine the role of this protein in the *S. mansoni* parasite biology and infection establishment.

## Materials and Methods

### Ethics Statement

All animals’ procedures were performed under Brazil national guidelines following Law 11794/08. Experiments were approved by the Ethics Commission on Animal Use (CEUA) of Oswaldo Cruz Foundation under numbers LW12/16 for hamsters and LM05/18 for mice.

### Primer Design

SmFES (Smp_332370) specific primers were designed based on the mRNA sequence predicted from *S. mansoni* genome (Version 7) available at WormBase^1^. The software Primer 3^2^ and GenScript Online PCR Primers Designs Tool^3^ were used for primer design. The T7 RNA polymerase promoter sequence (taatacgactcactataggg) was added on the 5′ end of the primers designed for dsRNA template amplification (Supplementary Table S1). The software OligoAnalyzer 3.1^4^ was used for secondary structures analysis.

### Synthesis of dsRNA

A fragment of 376 bp from SmFES transcript was amplified by PCR using GoTaq Green Master Mix (Promega) according to suppliers’ protocol. Cycle conditions were 40 cycles using 94°C for 30 s, 55°C for 30 s, and 72°C for 1 min. PCR products were separated on 1% agarose gel stained with ethidium bromide, purified using Wizard SV Gel and PCR Clean-Up System (Promega), and cloned into pGEM-T Easy vector (Promega). Then, plasmids containing the target insert were used to transform *Escherichia coli* DH5α. Plasmid DNA was extracted using the PureYield Plasmid Miniprep System (Promega) as manufacturer’s instructions, analyzed on 1% agarose gel, and purified as described above, in order to be used as a template for further PCR.

Afterward, specific dsRNAs were produced using T7 RiboMAX Express RNAi System (Promega) following manufacturers’ instructions. The green fluorescent protein (GFP) cloned into pCRII vector was used as a template for dsRNA synthesis of the unspecific control for RNAi. SmFES and GFP-dsRNA integrity were analyzed on 1% agarose gel stained with ethidium bromide and quantified at a NanoDrop 2000 (Thermo Fisher Scientific).

### Schistosomula Culture and dsRNA Exposure

Cercariae obtained from the Mollusk rearing facilities “Lobato Paraense” of René Rachou Institute, FIOCRUZ, were mechanically transformed into schistosomula (Milligan and Jolly, 2011). Then, 55,000 parasites were maintained in 6-well plates with 5 mL of Glasgow Minimum Essential Medium (GMEM) (Sigma-Aldrich) supplemented with 0.1% glucose (Vetec); 0.1% lactalbumin (Sigma-Aldrich); 20 mM of HEPES (Sigma-Aldrich); 2% inactivated fetal bovine serum (Gibco, Thermo Fisher Scientific); 0.5% MEM vitamin solution (Gibco, Thermo Fisher Scientific); 5% Schneider’s Insect Medium (Sigma-Aldrich); 0.2 μM of triiodothyronine (Sigma-Aldrich); 0.5 μM of hypoxanthine (Sigma-Aldrich); 1 μM of hydrocortisone (Sigma-Aldrich); and 1% penicillin/streptomycin (Gibco, Thermo Fisher Scientific). SmFES-dsRNA and GFP-dsRNAs were added to a final concentration of 200 nM, and the culture was incubated for ten days in a biological oxygen demand (BOD) incubator at 37°C under 5% CO_2_ and 95% humidity.

For viability analysis, 100 worms were removed daily and stained with 5 μg/mL of propidium iodide (Sigma-Aldrich) under a fluorescent inverted microscope Axio Vert (Zeiss) at 5× magnification and 544-nm wavelength. Images from culture were also obtained at 5× magnification for the area assessment using the software AxionVision 4.8 (Zeiss); for all experiments, three biological replicates were performed.

In order to evaluate schistosomula motility, 100 parasites from each experimental group were recorded, for 30 s for seven days using a fluorescent inverted microscope Axio Vert (Zeiss) at 5× magnification and the Debut Video Capture software^5^. Thus, videos were analyzed using the wrMTrck plugin in the ImageJ software^6^. Videos were converted to gray scale, and the Otsu threshold was applied. Then, the perimeter standard deviation was evaluated and used to determine schistosomulum movement. Only parasites that were detected in at least 35 frames of each video were considered in the analyses. A schematic representation of the methodology applied is depicted in Supplementary Figure S1.

To evaluate SmFES transcript levels after dsRNA exposure, 5,000 schistosomula were removed from culture every day for RNA extraction for seven days.

### Mice Infection and Parasitemia Evaluation

Three days after dsRNA exposure, female Swiss Webster mice were injected with 350 schistosomula from SmFES-dsRNA, GFP-dsRNA (unspecific control group), and untreated control groups (schistosomula non-exposed to dsRNAs). Infection was carried using 10 mice for each group, and five replicates were performed. After 42 days of infection, mice were euthanized with overdose following the animal facility of René Rachou Institute, FIOCRUZ, recommendations. For that, first, the animals were anesthetized with xylazine hydrochloride (10 mg/kg) (Syntec) and ketamine hydrochloride (150 mg/kg) (Syntec) by intramuscular administration, followed by 2.5% sodium thiopental (150 mg/kg) (Cristália). Then, perfusion to recovery adult worms was performed according to Pellegrino and Siqueira (1956).

Recovered adult worms were counted, and their length was measured, or they were subjected to RNA extraction for SmFES transcript level analysis. For adult worms’ size analysis, images from 30 worm pairs per animal were obtained under 1.2× magnification at Zeiss Microscope Stemi SV 11 (Zeiss). Parasites’ length was measured using ImageJ software^6^. Remaining adult worms recovered from mice were fixed in Alcohol-Formalin-Acetic Acid (AFA) solution containing 95% ethanol (Merck Millipore), 3% formaldehyde (Dinâmica), and 2% glacial acid acetic (Merck Millipore) and then stained for 30 min with 2.5% chloride carmine (Sigma-Aldrich). Thereafter, worms were washed with 70% ethanol (Merck Millipore) and quickly immersed in hydrochloric ethanol solutions containing 3% HCl (Synth) and 70% ethanol (Merck Millipore) followed by dehydration using 70%, 80%, 95%, and absolute ethanol (Merck Millipore), allowing 5 min for each concentration. Next, they were clarified in methyl salicylate (Dinâmica) with Canadian balsam (Synth) (1:2) for at least 24 h, and worms were individually mounted into slides. Confocal images of 30 females and eight males, per group, were captured using an inverted microscope Eclipse Ti-E (Nikon) with Confocal C2 plus (Nikon) under 10 and 40× magnification using 546- and 488-nm wavelengths. Confocal images were analyzed using NIS-Elements software (Nikon).

Mice intestine was removed, and 2–3 cm of the ileum was placed in microscope slides in order to assess the stages of egg maturation. The eggs from the first to fourth maturation stage were considered immature (Mati and Melo, 2013). Mice liver was also removed, and the median lobe was fixed in 4% buffered formaldehyde. The liver median lobes were embedded in paraffin, and tissue sections were stained with hematoxylin (Renylab) and eosin (Sciavicco). Granulomas were analyzed under a fluorescent inverted microscope Axio Vert (Zeiss) at 5× magnification, and images were acquired for granuloma area measurement using the ImageJ software^6^.

The remaining liver and intestine were separately weighed, sliced, and incubated in 10% potassium hydroxide overnight; and the eggs were counted under a light microscope Axiostar Plus (Zeiss), following a protocol previously described (Avelar et al., 2019). For fecundity analysis, the total number of eggs recovered from the liver and intestine of each animal was divided by the number of females adult worms recovered from the respective mouse. A schematic representation of the methodology applied is depicted in Supplementary Figure S1.

### Egg Culture and dsRNA Exposure

Female Golden hamsters were infected with 400 cercariae, and after 45 days of infection, euthanasia was performed as described above. The livers were removed, and egg extraction was made according to Rinaldi et al. (2009), with minor modifications. After liver digestion, the eggs were resuspended with 3 mL of Dulbecco’s modified Eagle’s medium (DMEM) (Sigma-Aldrich) supplemented with 1% penicillin/streptomycin (Gibco, Thermo Fisher Scientific), 80 μg/mL of gentamicin (Gibco, Thermo Fisher Scientific), and 10% inactivated fetal bovine serum (Gibco, Thermo Fisher Scientific).

Eggs were counted and incubated in 6-well plates (110,000 eggs per group) with 3 mL of DMEM supplemented and 20 μg/mL of dsRNA. Eggs soaked with SmFES-dsRNA and GFP-dsRNAs were cultivated, for seven days, in a BOD at 37°C under 5% CO_2_ and 95% humidity. Four replicates were performed.

During incubation, at the third, fifth, and seventh days, 20,000 eggs were removed for RNA extraction. On the seventh day, the remaining eggs were transferred to 1.5-mL tubes and washed three times with phosphate-buffered saline (PBS). Then, the wash was repeated using dechlorinated tap water, and the eggs were exposed to the light for 30 min for miracidia hatching. After light exposure, miracidia capacity to infect *B. glabrata* (Mollusca: Planorbidae) and the effect of snail mucus on miracidial behavior were evaluated. Additionally, 100 eggs were placed in 96-well plates, exposed to light, and fixed in 4% formaldehyde. The hatching percentage was evaluated under the microscope Axiostar Plus (Zeiss).

For mucus-attraction evaluation, assays were performed as described by Wang et al. (2019), with minor modifications. Miracidia were placed in 6-well plates with 1 mL of water without chlorine. Miracidia movement was recorded for 1 min in a Zeiss Microscope Stemi SV 11 (Zeiss) using a 12-megapixel camera with 30 frames per second, under 10× magnification. Then, 70 μL of mucus from *B. glabrata* was added, and the miracidial behavior was recorded for one additional minute. Miracidia trajectories and velocity, before and after mucus addition, were analyzed using Tracker Video Analysis and Modeling Tool version 5.1.2^7^.

For *B. glabrata* challenge, 30 snails were individually exposed to eight miracidia. Miracidia penetration was evaluated one and four hours after exposure. After evaluation, snails were maintained at an aquarium with lettuce *ad libitum*.

After 28 and 42 days post challenge, snails were individually exposed to light for 40 min. The number of cercariae released per snail (infection intensity) was estimated in three aliquots of 100 μL, and the RNA of released cercariae was extracted.

Female Swiss Webster mice were intraperitoneally infected with released cercariae. Animals were first anesthetized with xylazine hydrochloride (10 mg/kg) (Syntec) and ketamine hydrochloride (150 mg/kg) (Syntec). Mice were immobilized, and 100 cercariae were placed over the shaved abdomen during 1 h (Smithers and Terry, 1965). For each group, five animals were used, and two biological replicates were performed. After 42 days, animals were euthanized; and perfusion for eggs and adult worms’ recovery was performed. Adult worms’ length from two worm pairs per animal were measured as previously described. A schematic representation of the methodology applied is depicted in Supplementary Figure S1.

### Reverse Transcriptase Quantitative PCR (RT-qPCR)

For SmFES transcript levels analyses, total RNA of schistosomula, adult worms, eggs, and cercariae samples were extracted. First, parasites were homogenized and lysed using TRIzol reagent (Invitrogen), and procedures until phase separation step were performed following manufacturers’ protocol. Then, for RNA isolation, the aqueous phase was placed into columns from SV Total RNA Isolation System (Promega), and RNA was obtained according to suppliers’ protocol. The remaining genomic DNA was removed using the Turbo DNase (Ambion). The total RNA was quantified using Qubit RNA HS Assay Kit (Invitrogen) at a Qubit 2.0 Fluorometer (Invitrogen). The cDNA was synthesized using ImProm-II Reverse Transcription System (Promega).

Primer concentration to reach the maximum quantitative PCR (qPCR) efficiency was standardized according to Bustin et al. (2009) using the ViiA 7 system (Applied Biosystems). Reactions were performed in three technical replicates. Cytochrome *c* oxidase subunit I (COX1, Smp_900000) from *S. mansoni* was used as an endogenous control for qPCR. For each sample was used 10 ng/μL of RNA synthesized in cDNA, 1 × SYBR Green PCR Master Mix (Applied Biosystems), 900 nM of each forward and reverse primers for SmFES and 400 nM for COX1 at 10 μL of final volume. SmFES transcript levels from parasites exposed to SmFES-dsRNA were compared with those in parasites exposed to GFP-dsRNA and untreated control parasites. Relative expression was analyzed using the ΔΔCt method (Livak and Schmittgen, 2001).

Absolute quantification was performed to evaluate Sm*fes* expression among *S. mansoni* stages (miracidia, sporocysts, cercariae, two and seven days’ schistosomula, and male and female adult worms). Miracidia and cercariae were kindly provided by the Mollusk rearing facility “Lobato Paraense” of the René Rachou Institute, FIOCRUZ. Schistosomula and adult worms were obtained as described. Sporocysts used in this assay were transformed after 24- to 48-h incubation of miracidia in Chernin’s balanced saline solution, in a BOD at 26°C, for the transformation into mother sporocysts (Laursen and Yoshino, 1999). A synthetic SmFES sequence cloned into a pCold-GST vector (Takara Bio) was obtained from GenScript, and a dilution curve of pCold-GST-SmFES was made using five dilutions from 1 × 10^9^ to 1 × 10^1^ copy numbers. Reactions were performed in triplicate as previously described. After the amplification, the absolute copy number was calculated by interpolation between the Cts of pCold-GST-SmFES dilution curve and Cts from each *S. mansoni* stage (Lee et al., 2006).

### Statistical Analyses

All statistical analyses were run using GraphPad Prism 8. Normality test using the Shapiro–Wilk test was previously applied to all generated data.

Mann–Whitney test was used to analyze transcript levels, data from egg maturation assessment, miracidia speed, mucus attraction, adult worms’ length, and area of ovary. Analyses of schistosomula and granuloma area, miracidia penetration, recovering of adult worms and eggs from liver and intestine, and female adult worms’ fecundity were performed using unpaired *t*-test. Two-way ANOVA with Sidak’s multiple comparisons tests were used to analyze schistosomula mortality. All analyses were made relative to untreated and unspecific control groups. Differences were considered significant when *P*-value < 0.05.

### Reagents

The catalog number of reagents used in this work is as follows. Promega: GoTaq Green Master Mix (M7122), Wizard SV Gel and PCR Clean-Up System (A9282), pGEM-T Easy vector (A1360), PureYield Plasmid Miniprep System (A1222), T7 RiboMAX Express RNAi System (P1700), SV Total RNA Isolation System (Z3105), and ImProm-II Reverse Transcription System (A3800). Sigma-Aldrich: GMEM (G6148), lactalbumin (L7252), HEPES (H3375), triiodothyronine (709611), hypoxanthine (H9636), hydrocortisone (H0888), Schneider’s Insect Medium (S0146), propidium iodide (P4170), Dulbecco’s modified Eagle’s medium (D5030), and carmine (C1022). Gibco, Thermo Fisher Scientific: fetal bovine serum (16000044), MEM vitamin solution (11120052), penicillin/streptomycin (15070063), and gentamicin (15710064). Applied Biosystems: SYBR Green PCR Master Mix (4309155). Ambion: Turbo DNase (AM2238). Invitrogen: Qubit RNA HS Assay Kit (Q32855). Syntec: xylazine hydrochloride (v-007) and ketamine hydrochloride (v-001). Cristália: sodium thiopental (22.1535). Renylab: hematoxylin (0012). Sciavicco: eosin (EA36). Vetec: Glucose (V000221. Synth: Canadian balsam (00B1001.08.BJ). Merck Millipore: ethanol (64-17-5), glacial acid acetic (64-19-7), and HCl (109057). Dinâmica: formaldehyde (P.10.0504.000.000) and methyl salicylate (60READIN016786).

## Results

### Sm*fes* Expression Is Higher in *Schistosoma mansoni* Free-Living Stages

**FIGURE 1:**
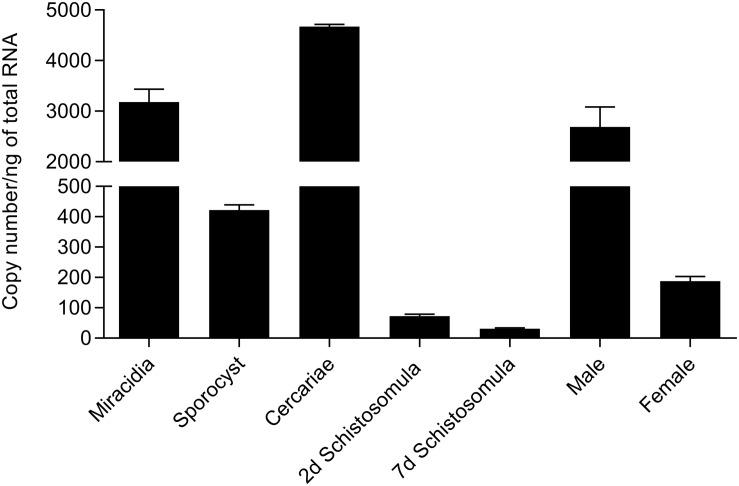
Assessment of Sm*fes* expression among *Schistosoma mansoni* life stages. Bar graph representing absolute SmFES transcript levels in copy numbers per nanogram of total RNA in miracidia, sporocyst, cercariae, 2 and 7 days’ schistosomula, and male and female adult worms. Bars represent the mean of three technical replicates. Error bars are represented above the bars.

SmFES transcript expression among the different *S. mansoni* life stages was assessed by RT-qPCR (Figure 1). Cercariae and miracidia free-swimming stages presented higher Sm*fes* expression than the other stages, accounting for 4,670.5 and 3,182.1 copies/ng of total RNA, respectively. Schistosomula and female adult worms presented the lowest Sm*fes* expression levels: 31.3 copies/ng for seven days’ schistosomula, 72.6 copies/ng for two days’ schistosomula, and 187.9 copies/ng for female adult worms. Sporocysts and male adult worms presented 421.9 and 2,691.9 copies/ng, respectively.

### Sm*fes* Knockdown Affects Schistosomula Size and Viability

**FIGURE 2:**
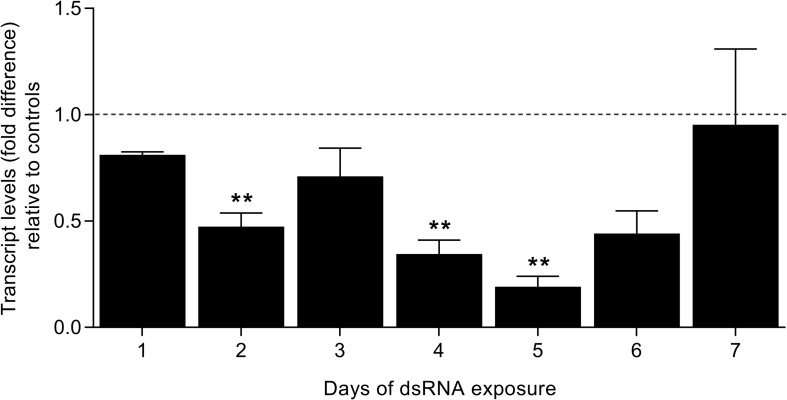
Evaluation of SmFES transcript levels in schistosomula exposed to SmFES-dsRNA for 7 days. Bar graph representing the mean of SmFES transcript levels in schistosomula exposed to SmFES-dsRNA for 7 days. The dotted line represents normalized SmFES transcript levels in parasites exposed to green fluorescent protein (GFP)-dsRNA and in the untreated control. Error bars are represented above the bars. Statistical analysis using Mann–Whitney test is represented with asterisks above the bars (**P < 0.01).

In order to verify the roles of SmFES in schistosomula, the gene was knocked-down with dsRNA, and the transcript levels were verified for seven days by RT-qPCR. Parasites showed a reduction in transcripts starting on the second day of exposure (52%). After five days, schistosomula presented an 80.8% reduction in SmFES transcript levels. A statistically significant reduction in transcript levels was observed on the second, fourth (65.5%), and fifth days of SmFES-dsRNA exposure when compared with control groups (*P* < 0.01) (Figure 2).

**FIGURE 3:**
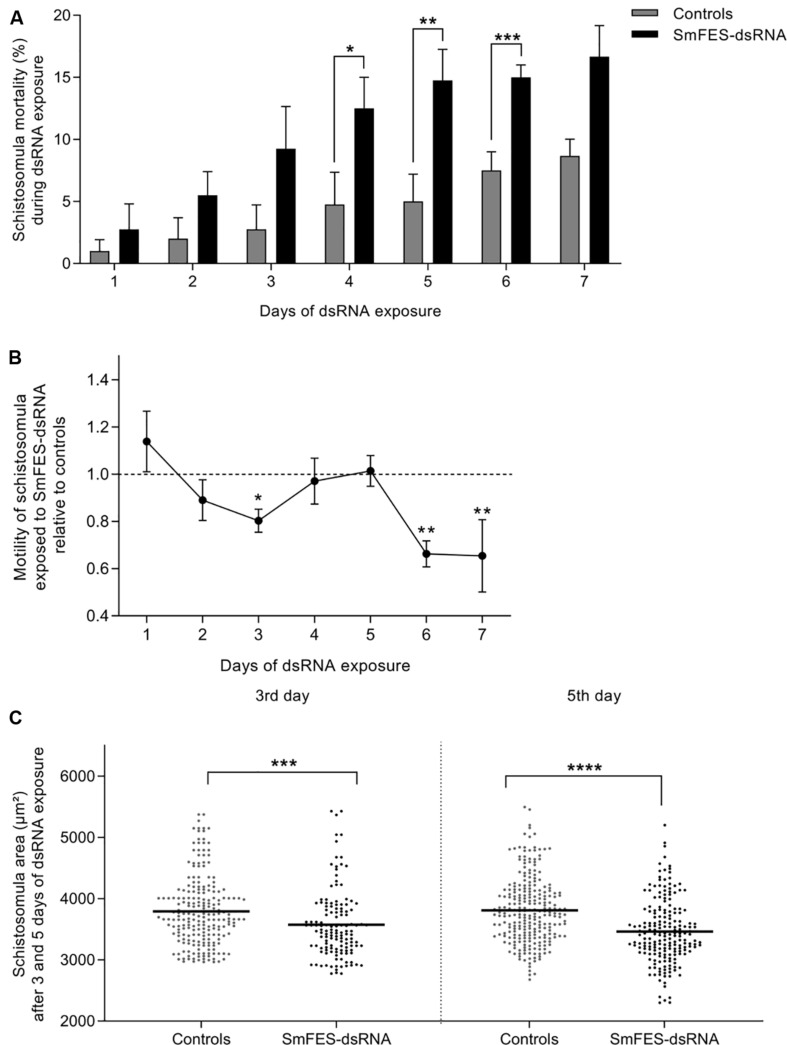
Phenotypic analyses of schistosomula exposed to SmFES-dsRNA *in vitro*. **(A)** Bar graph representing the mean (%) of schistosomula mortality from control groups (gray) or exposed to SmFES-dsRNA (black) for 7 days. Error bars are represented above the bars. Statistical analyses using two-way ANOVA with Sidak’s multiple comparisons test are represented with asterisks above the bars (*N* = 4, *P < 0.05, **P < 0.01, ***P < 0.001). **(B)** Graph showing the motility mean of schistosomula exposed to SmFES-dsRNA during 7 days normalized with the data from untreated control and parasites exposed to GFP-dsRNA (Controls). Error bars are represented in each symbol, and the dotted line represents the control groups. Statistical analyses using two-way ANOVA with Sidak’s multiple comparisons test are represented with asterisks above the symbols (*N* = 2, *P < 0.05, **P < 0.01). **(C)** Area (mm^2^) of schistosomula from untreated control and exposed to GFP-dsRNA (controls; gray) or SmFES-dsRNA (black) for 3 (left) and 5 days (right). Each dot represents the area of one schistosomulum, and the horizontal black line represents the median. Statistical analysis using unpaired *t*-test is represented with asterisks (***P < 0.001, ****P < 0.0001).

Schistosomula exposed to SmFES-dsRNA presented higher mortality *in vitro* when compared to untreated and unspecific control groups from the fourth day of exposure. Mortality remained significant (*P* < 0.05) until the sixth day, ranging from 10 to 20% (Figure 3A). After motility analysis, a reduction of 19.7% in the schistosomula movement on the third day of SmFES-dsRNA exposure (*P* < 0.05) was observed. On the sixth and seventh days, a motility alteration was also verified, presenting a decrease of 33.7 and 34.5%, respectively (*P* < 0.01) (Figure 3B).

The area of knocked-down parasites was measured in order to detect possible phenotype alterations. We observed a size reduction of 7% in the Sm*fes*-knocked-down group when compared with control groups three days after dsRNA exposure (*P* < 0.001). Additionally, schistosomula exposed to SmFES-dsRNA for five days were 10% smaller than schistosomula from control groups (Figure 3C) (*P* < 0.0001).

### Sm*fes* Knockdown in Schistosomula Influences the Establishment of Mammalian Infection

To elucidate whether SmFES is important during mammalian infection establishment, mice were inoculated with Sm*fes*-knocked-down schistosomula. As great mortality of schistosomula exposed to SmFES-dsRNA was observed from the fifth day, we chose to perform mouse infection using three days’ schistosomula to avoid inoculation of dead parasites, because it could influence the number of recovered adult worms.

**FIGURE 4:**
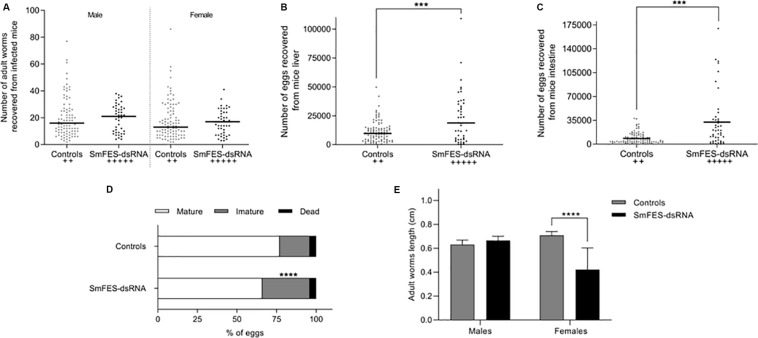
Schistosomula Sm*fes* knockdown effects after mammalian infection. Dispersion graph representing the number of adult worms males (left) and females (right) **(A)** and eggs from the liver **(B)** and intestine **(C)** recovered from mice after 42 days of infection with schistosomula from untreated and green fluorescent protein (GFP) control groups (controls, gray) or exposed to SmFES-dsRNA (black) for 3 days. Each dot represents the number of worms/eggs recovered from one mouse. Median is represented by the horizontal black line. Statistical analysis using unpaired *t*-test with Welch’s correction is represented with asterisks (***P < 0.001). The cross symbols represent dead mice. **(D)** Bar graph representing the percentage of mature (white), immature (gray), and dead (black) eggs in the mice ileum after 42 days of infection. Statistical analysis of immature egg numbers using Mann–Whitney test is represented with asterisks above the bars (****P < 0.0001). **(E)** Bar graph representing the median length of adult worms recovered from mice infected with schistosomula from control groups (gray bar) or Sm*fes*-knocked-down schistosomula (black bar). Above the bars are represented the 95% confidence interval. Statistical analysis using Mann–Whitney test is represented with asterisks (****P < 0.0001).

Mice infected with schistosomula exposed to SmFES-dsRNA presented 30.7 and 31.25% more females and males adult worms, respectively, when compared to control groups (Figure 4A). Moreover, a significant increase in eggs recovered from the liver (96%) and intestine (237%) was also observed in the SmFES group (*P* < 0.001) (Figures 4B,C). Fecundity of SmFES females was also higher than in females from control groups (Supplementary Figure S2A).

Further, the stages of maturation of eggs derived from the mice ileum were also analyzed. We observed a 57% increase of immature eggs in mice infected with Sm*fes*-knocked-down schistosomula in comparison with mice from control groups (*P* < 0.0001) (Figure 4D).

The length of recovered adult worms was measured, and no difference between the size of males from controls and Sm*fes*-knocked-down groups was observed. Yet the length of females recovered from mice infected with Sm*fes*-knocked-down schistosomula presented a 40.4% decrease (*P* < 0.0001) when compared with the size of females recovered from mice infected with untreated and GFP-dsRNA-exposed schistosomula (Figure 4E and Supplementary Figure S2B).

**FIGURE 5:**
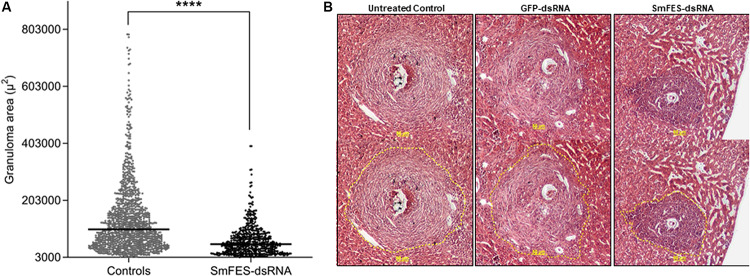
Analysis of granuloma from liver of mice infected with Sm*fes*-knocked-down schistosomula. **(A)** Area (μm^2^) of hepatic granulomas from mice after 42 days of infection with schistosomula from control groups (gray) or exposed to SmFES-dsRNAs (black) for 3 days. Each dot represents the area of one granuloma. Median is represented by the horizontal black line. Statistical analysis using unpaired *t*-test with Welch’s correction is represented with asterisks (****P < 0.0001). **(B)** Representative hematoxylin and eosin-stained liver sections from the untreated control group or exposed to green fluorescent protein (GFP)- or SmFES-dsRNAs. Yellow bars indicate 50 μm, and the dashed yellow lines delimit the area measured by ImageJ.

Despite the increase of the number of eggs found in mice tissues, granulomas from the median lobe were analyzed, and a 48% reduction (*P* < 0.0001) in granuloma area was observed in livers from mice infected with Sm*fes*-knocked-down schistosomula when compared with granulomas from livers of control groups (Figures 5A,B).

### Sm*fes* Knockdown in Schistosomula Alters the Adult Worms’ Reproductive System

Confocal analyses of adult worms recovered from mice infected with Sm*fes*-knocked-down schistosomula were conducted to evaluate alterations in the worms’ development and maturation.

**FIGURE 6:**
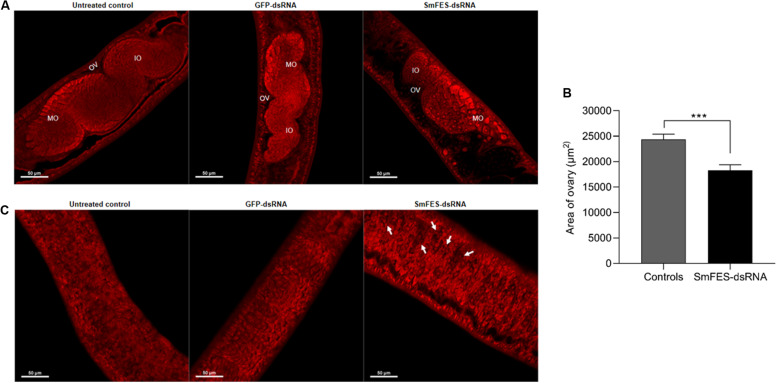
Analysis of ovary and vitellarium of females recovered from mice infected with Sm*fes*-knocked-down schistosomula. **(A)** Representative confocal images showing the ovary of females recovered from mice infected with schistosomula from untreated control or exposed to green fluorescent protein (GFP)- or SmFES-dsRNAs for 3 days. White bars indicate 50 μm. Abbreviations: OV, ovary; IO, immature oocyte; MO: mature oocyte. **(B)** Bar graph representing the median area of females’ ovary recovered from mice infected with schistosomula from control groups (gray bar) or Sm*fes*-knocked-down schistosomula (black bar). Above the bars are represented the 95% confidence interval. Statistical analysis using Mann–Whitney test is represented with asterisks (***P < 0.001). **(C)** Representative confocal images showing the vitellarium of females recovered from mice infected with schistosomula from untreated control or exposed to GFP- or SmFES-dsRNAs for 3 days. Arrows show the holes in the vitellarium, and white bars indicate 50 μm.

The ovary of females recovered from mice infected with Sm*fes*-knocked-down schistosomula presented no changes in the number and distribution of mature or immature oocytes (Figure 6A), although the ovary presented an area 24% smaller (*P* < 0.001) when compared with the ovary from the control groups (Figures 6A,B). After the analyses of the vitellaria, it was observed that females from SmFES group exhibited holes in this organ, in contrast to the vitellaria from untreated and GFP-dsRNA groups (Figure 6C).

The ootype from females and testicular lobes from males presented no alterations among experimental and control groups (Supplementary Figures S3A,B).

### Sm*fes* Knockdown in *Schistosoma mansoni* Eggs Changes Miracidial Behavior

Considering that Sm*fes* expression is high in the free-living miracidium stage and an increase in immature eggs was noticed in the intestine from mice infected with Sm*fes*-knocked-down schistosomula, we sought to understand SmFES roles in *S. mansoni* eggs/miracidia.

**FIGURE 7:**
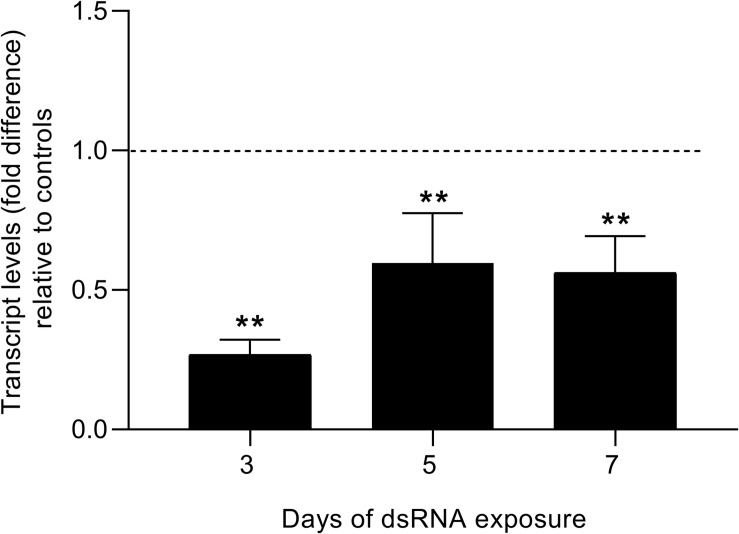
Evaluation of SmFES transcript levels in eggs exposed to SmFES-dsRNA for 3, 5, and 7 days. Bar graph showing SmFES transcript levels mean in eggs exposed to SmFES-dsRNA during 3, 5, and 7 days. The dotted line represents normalized SmFES transcript levels in eggs from control groups. Error bars are represented above the bars. Statistical analysis using Mann–Whitney test is represented with asterisks above the bars (*N* = 3, **P < 0.01).

Sm*fes* was successfully knocked-down in eggs from the third day after dsRNA exposure, presenting a 73% reduction of transcript levels when compared with unspecific and untreated controls (Figure 7). The decrease in Sm*fes* expression remained after seven days of dsRNA exposure with eggs presenting 44% less SmFES transcripts. The reduction in transcripts observed along the analyzed days was statistically significant (*P* < 0.001).

**FIGURE 8:**
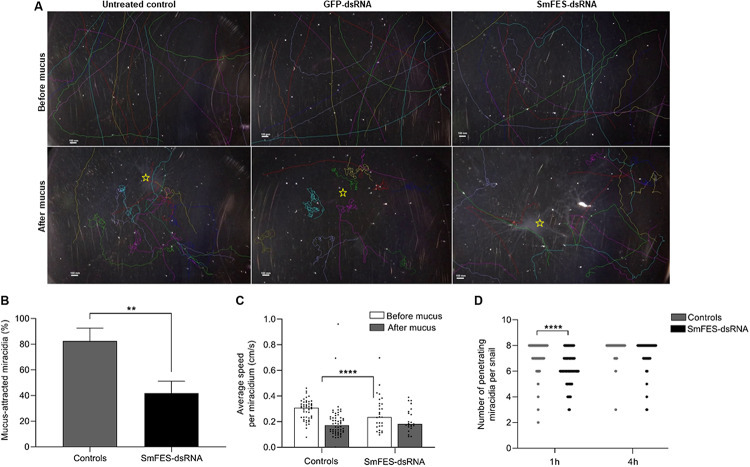
Behavior assessment of miracidia hatched from Sm*fes*-knocked-down eggs. **(A)** Images showing miracidia route before (above) and after (below) mucus addition in plates containing miracidia hatched from untreated control eggs or exposed to green fluorescent protein (GFP)- or SmFES-dsRNA. Each line represents the route of one miracidium. **(B)** Bar graph shows the percentage of miracidia attracted to *Biomphalaria glabrata* mucus from control groups (gray) or Sm*fes*-knocked-down (black). Error bars are represented above the bars. Statistical analysis using Mann–Whitney test is represented with asterisks (**P < 0.01). **(C)** Bar graph representing the average speed (cm/s) of each miracidium hatched from control groups or Sm*fes*-knocked-down eggs before (white) and after (gray) mucus addition. Each symbol represents the speed of one miracidium, and bars represent the median. Statistical analysis using Mann–Whitney test is represented with asterisks (****P < 0.0001). **(D)** Bar graph depicting the median number of penetrating miracidia per snail 1 and 4 h after *B. glabrata* challenge with miracidia hatched from the control groups (gray) or Sm*fes*-knocked-down eggs (black). Statistical analysis using unpaired *t*-test with Welch’s correction is represented with asterisks (****P < 0.0001).

After the knockdown, we analyzed the miracidia hatching ability. No difference was observed in the number of hatched miracidia among the controls and Sm*fes*-knocked-down eggs (Supplementary Figure S4). To identify miracidial behavior alterations, parasites were recorded before and after the addition of *B. glabrata* mucus. After miracidia migration tracking, it was observed that following mucus addition, miracidia from untreated and unspecific control groups swam toward the mucus and started a continuous circular movement at the spot where the mucus was added. In contrast, most of the miracidia from Sm*fes*-knocked-down eggs presented no change after mucus addition (Figure 8A and Supplementary Movies S1–S3). Mucus attraction was quantified, and we observed that miracidia from Sm*fes*-knocked-down group showed 51% less mucus attraction than the untreated and unspecific control groups (*P* < 0.01) (Figure 8B). Parasites’ speed was also measured, and while the control groups showed a 56% speed reduction after the mucus addition (*P* < 0.0001), miracidia hatched from Sm*fes*-knocked-down eggs did not present significant changes in their velocity (Figure 8C).

In order to check the capability of Sm*fes*-knocked-down miracidia to penetrate the intermediate host, *B. glabrata* were challenged with miracidia hatched from untreated and unspecific controls and Sm*fes*-knocked-down eggs. The number of miracidia able to penetrate the snail was counted after one and four hours. We observed that parasites from the SmFES group took longer to penetrate the snails. After 1 h of challenge, most of the parasites from control groups had already entered the snails, whereas in the SmFES group, 25% of the miracidia were unable to penetrate (Figure 8D). This difference was statistically significant (*P* < 0.0001). After 4 h, it was observed that 18.7% of Sm*fes*-knocked-down miracidia were incapable to penetrate the host, whereas parasites from control groups successfully penetrated the snails.

### SmFES RNAi Effect in Miracidia Generates Knocked-Down Cercariae and Affects the Female Worm Development and Reproduction in the Mammalian Host

To verify whether Sm*fes*-knocked-down miracidia were able to normally develop within the intermediate host, the challenged snails were exposed to light for cercaria release analysis after 28 and 42 days. Cercariae released from each infected snail was counted, and there was no difference among mollusks challenged with miracidia from eggs exposed to SmFES-dsRNA and miracidia hatched from unspecific and untreated control groups (Supplementary Figure S5).

**FIGURE 9:**
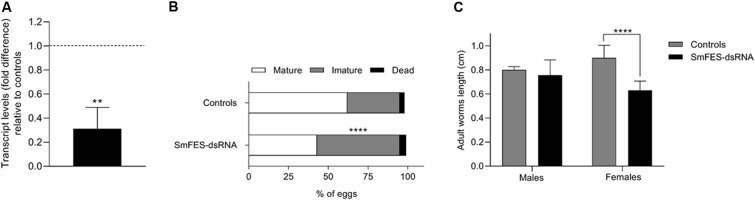
Evaluation of SmFES transcript levels of cercariae derived from Sm*fes*-knocked-down eggs and the effects in the mammalian host infection outcome. **(A)** Bar graph depicting SmFES transcript levels mean in cercariae released from snails exposed to miracidia hatched from Sm*fes*-knocked-down eggs. The dotted line represents normalized SmFES transcript levels in cercariae released from snails exposed to miracidia from control groups. Error bar is represented above the bars. Statistical analysis using Mann–Whitney test is represented with asterisks (*N* = 2, **P < 0.01). **(B)** Bar graph representing the percentage of mature (white), immature (gray), and dead (black) eggs in mice ileum after 42 days of cercariae infection. Statistical analysis of immature egg numbers using Mann–Whitney test is represented with asterisks above the bars (****P < 0.0001). **(C)** Bar graph representing the median length of adult worms recovered from mice infected with cercariae from controls group (gray) and from mice infected with Sm*fes*-knocked-down cercariae (black). Above the bars are represented the 95% confidence interval. Statistical analysis using Mann–Whitney test is represented with asterisks (****P < 0.0001).

Analysis of Sm*fes* expression in released cercariae showed that parasites from snails infected with miracidia hatched from SmFES-dsRNA-exposed eggs remained with decreased transcript levels, presenting 68.7% less (*P* < 0.01) transcripts when compared with controls (Figure 9A).

Mice infected with Sm*fes*-knocked-down cercariae presented 24.4% more male adult worms (*P* < 0.05) (Supplementary Figure S6A) and a 75.6% increase (*P* < 0.05) of eggs from the intestine relative to control groups (Supplementary Figure S6C). No difference in the number of recovered female adult worms and eggs from the liver was observed (Supplementary Figures S6A,B). Also, a 53.8% increase of immature eggs (*P* < 0.0001) was found in the ileum of mice infected with Sm*fes*-knocked-down cercariae relative to control groups (Figure 9B). Additionally, female adult worms recovered presented a 29.9% length decrease (*P* < 0.0001) when compared with females recovered from mice infected with cercariae from control groups (Figure 9C and Supplementary Figure S6D).

## Discussion

The present work demonstrates that Sm*fes* expression levels are higher in cercariae and miracidia of *S. mansoni*, which could reflect its importance in the free-living life stages of the parasite. Our data corroborate with previous studies that indicate that Sm*fes* is more expressed in cercariae than in other stages (Bahia et al., 2007; Logan-Klumpler et al., 2012) and present lower expression in schistosomula 48 h (Ludolf et al., 2007) and seven days after transformation (Bahia et al., 2007; Logan-Klumpler et al., 2012), although it is divergent from what was found in RNA-Seq analyses in which Sm*fes* presented higher expression in males and females adult worms (Lu et al., 2017).

SmFES TK seems to be important to schistosomula survival *in vitro* because there was rising mortality of schistosomula exposed to SmFES-dsRNA, which was reinforced by the motility decrease and alterations in size observed. In *Caenorhabditis elegans* (Nematoda: Rhabditidae), the knockdown of Fer, which is a TK from FES protein family, indicated that this protein has a role in the nematode epidermal differentiation (Putzke, 2005). Additionally, the human FES ortholog presents a role in actin and tubulin cytoskeleton regulation (Laurent and Smithgall, 2004; Laurent et al., 2004); therefore, the phenotypic alterations in schistosomula might be due to filament rearrangements. Those results indicate that SmFES is important for schistosomula growth and survival *in vitro*.

Despite that the number of adult worms recovered from mice from Sm*fes*-knocked-down schistosomula presented no significant difference, the decrease of Sm*fes* expression alters the outcome of infection in the mammalian host. An increased number of eggs from livers and intestines of mice infected with Sm*fes*-knocked-down schistosomula was observed; thus, parasite fecundity was higher. However, an elevated rate of immature eggs from the SmFES group was seen. As Sm*fes* is more expressed in the miracidium stage, the Sm*fes* suppression could influence embryo development. Also, soluble egg antigen (SEA) released by eggs mediate immunological responses during egg migration through the host tissues (Lenzi et al., 1987; Doenhoff et al., 2003). Mature eggs secrete more SEA than immature eggs, and the molecules responsible for triggering host immune responses are secreted only when the miracidium is completely formed, which, in turn, allows eggs to discharge outside (Ashton et al., 2001; Pearce and MacDonald, 2002; Schramm et al., 2006). As we found more immature eggs in the ileum of mice infected with Sm*fes*-knocked-down schistosomula, it could be that these eggs were not capable of expressing or secreting proteins that would trigger the host immune responses to promote egg release to the external environment. This might explain the increased number of eggs trapped in the mammalian host tissues.

The vitellarium and ovary are organs from the reproductive system of the female adult worm and are essential to produce viable eggs (Clough, 1981). Adult worm mating status is essential for female maturation because the differentiation processes of the ovary and vitellarium are complete as a result of the pairing (Erasmus, 1973; Kunz, 2001; LoVerde et al., 2004). The contact with the male adult worm also affects female size, which also affects the contact with the male adult worm, because non-paired females present a reduced size (Clough, 1981). For this reason, the mating also reflects in the oviposition because viable eggs are only produced by complete mature females (Shaw, 1987). Additionally, *S. mansoni* CTKs are described as probably involved in the differentiation process of the parasite reproductive system because they are described as highly expressed in adult worm gonads (Beckmann et al., 2010). However, SmFES role in this differentiation process was unknown. Here, we presented that females recovered from mice infected with Sm*fes*-knocked-down schistosomula exhibited a size reduction, smaller ovary, and holes in the vitellarium, indicating that these worms did not reach complete maturation (Erasmus, 1973; Popiel et al., 1984; Kunz, 2001; Michel et al., 2003). Some immature females can produce eggs as a result of a failure in the control mechanisms activation, but they are not viable (Shaw, 1987). Thus, the rate of immature eggs observed in the ileum of mice infected with Sm*fes*-knocked-down schistosomula may be due to the presence of immature females.

Egg release is facilitated by the intestine granuloma formation (Fallon et al., 2000; Schwartz and Fallon, 2018). This inflammation is also mediated by secreted SEA from mature eggs and immunomodulatory molecules from the eggshell, which induce a Th2 response (Sabin and Pearce, 1995; Hernandez et al., 1997; Cheever et al., 2000; Schwartz and Fallon, 2018). Thus, schistosome eggs are considered the main cause of schistosomiasis pathology and subsequent morbidity (Schwartz and Fallon, 2018). When deposited in the host tissues, egg antigens induce a T CD4+ immune response (Fallon et al., 1998; Boros, 1989), and then tumor necrosis factor-alpha (TNF-α) and intercellular adhesion molecule 1 (ICAM-1) expression contribute to lymphocyte activation for granuloma formation (Lukacs et al., 1994). Thus, the host immune responses are dependent on parasite stimulus. Although further investigations regarding immunomodulatory molecules triggered by this signaling pathway are necessary, SmFES may play a role in egg/miracidium maturation and/or participates in signaling pathways responsible for mediating SEA secretion or eggshell protein expression. This could result in a significantly smaller granuloma area in the liver recovered from mice infected with Sm*fes*-knocked-down parasites.

Further, because the Sm*fes* is highly expressed in miracidia and proved to have a role in embryo development, we performed Sm*fes* knockdown in eggs to evaluate miracidia hatching, motility, and capability to infect the intermediate host. After Sm*fes* knockdown, no difference in parasite hatching was noted. Meanwhile, miracidia derived from Sm*fes*-knocked-down eggs presented lower attraction to *B. glabrata.* Invertebrate host attraction is mediated by miracidium chemoreceptors responsible for sighting the snail-secreted chemicals in the mucus (Samuelson et al., 1984; Haas et al., 1995; Sukhdeo and Sukhdeo, 2004). When miracidia find the snail-secreted molecules, the swimming behavior changes from a straight line to a circular random movement around the mucus (MacInnis, 1965); this behavior was observed for miracidia from control groups, whereas most Sm*fes*-knocked-down miracidia remained with the same behavior observed before mucus addition. So Sm*fes* knockdown plays a role in sensory modulation.

Once the miracidium finds the invertebrate host, penetration lasts for about 15 min; and during the process, host sighting, terebratorium secretions, and rotational movements are very important for successful infection (Machado-Silva et al., 2008). Whereas miracidia from control groups took 1 h to penetrate the snail, Sm*fes*-knocked-down parasites required between one and four hours to complete *B. glabrata* invasion, yet some miracidia were incapable of penetrating the host. Those results and the Sm*fes* abundant expression in miracidium terebratorium (Bahia et al., 2007) suggest that this TK is also involved in the invertebrate host invasion. Within the snail host, the miracidium develops in sporocysts, replicates asexually, and differentiates in cercariae (Colley et al., 2014). The cercariae released from snails infected with Sm*fes*-knocked-down parasites showed a persisting reduction of SmFES transcript levels. Inheritance of gene knockdown promoting RNAi maintenance is reported in *C. elegans* (Vastenhouw et al., 2006). In this nematode, reports show that the progeny from knocked-down worms presents full transcript level reduction and can continue for successive generations (Burton et al., 2011; Marré et al., 2016; Spracklin et al., 2017). In *S. mansoni*, persistent knockdown has only been shown once, in which adult worms, recovered from mice inoculated with Smp38-knocked-down schistosomula, still present reduction in Smp38 transcript levels (Avelar et al., 2019). To our knowledge, this is the first time that the RNAi effect in eggs is kept stable during three life stages of *S. mansoni*, maintaining lower transcript levels to cercaria stage. After infecting mice with cercariae recovered from snails infected with Sm*fes*-knocked-down miracidia, we did not find significant differences in the recovery of worms and eggs from tissues, but there was a high impact in eggs and female worm maturation. These phenotypic alterations were similar to those observed after mouse infection with Sm*fes*-knocked-down schistosomula. So regardless of the stage in which the dsRNA was delivered, SmFES proved to be essential for *S. mansoni* development inside the mammalian host.

Additionally, human Fer and FES are described in tubulin and actin rearrangement and as part of the p38 signaling MAP kinase activation, promoting chemotaxis and cell migration (Craig and Greer, 2002; Craig, 2012). In *S. mansoni*, SmFES might participate in the activation of Smp38 signaling events because Smp38 is involved in the regulation of tubulin expression (Avelar et al., 2019) and miracidium ciliary beating regulation (Ressurreição et al., 2011). However, this is yet to be elucidated.

The current study presents, for the first time, the SmFES TK function in different *S. mansoni* life stages, *in vitro* and *in vivo*. Because granulomatous lesions are the main cause of schistosomiasis morbidity (Olveda et al., 2017) and considering the infection outcome, generating reduced granulomas in the livers, SmFES should be explored to aid disease morbidity control. Moreover, interrupting the snail infection is considered a complementary approach for schistosomiasis control (Gray et al., 2010; Ross et al., 2017), as SmFES seems to play a role in miracidia attraction to snails and infectivity regulation; it could impact the success of invertebrate host infection. There are long paths to be performed, but as specific molluscicides are extremely necessary to control the transmission in endemic areas, strategies based on stable soluble compounds (Augusto et al., 2017), which are able to specifically bind to SmFES, could enable the interruption of *Schistosoma* life cycle. Considering that, a reasonable approach could use an SmFES inhibitor in a restrained sewage treatment to prevent miracidium hatching and reduce mollusk infection. Therefore, SmFES inhibitors should be investigated to support schistosomiasis control.

## Data Availability Statement

All datasets generated for this study are included in the article/Supplementary Material.

## Ethics Statement

The animal study was reviewed and approved by Brazil national guidelines following Law 11794/08. Ethics Commission for Animal Use (CEUA) of Oswaldo Cruz Foundation under the numbers LW12/16 for hamsters and LM05/18 for mice.

## Author Contributions

NT, SG, and MM contributed to the conception and design of the study. NT, SG, GT, CP, BM, FL, LM, RC, and MM performed the experiments. NT performed the statistical analyses. LM and MM contributed reagents, materials, and analysis tools. NT, SG, and MM wrote the manuscript. All authors contributed to manuscript revision and read and approved the submitted version.

## Conflict of Interest

The authors declare that the research was conducted in the absence of any commercial or financial relationships that could be construed as a potential conflict of interest.

## Footnotes

1 https://parasite.wormbase.org/Schistosoma_mansoni_prjea36577

2 http://bioinfo.ut.ee/primer3-0.4.0/

3 https://www.genscript.com/tools/pcr-primers-designer

4 https://www.idtdna.com/calc/analyzer

5 https://www.nchsoftware.com/capture/index.html

6 https://imagej.nih.gov/ij/

7 https://physlets.org/tracker/
