# Supplementary material for: Schistosoma mansoni FES Tyrosine Kinase Involvement in the Mammalian Schistosomiasis Outcome and Miracidia Infection Capability in Biomphalaria glabrata
Source: Front Microbiol. 2020 Jun 11;11:963. doi: 10.3389/fmicb.2020.00963 (PMC7300192; doi:10.3389/fmicb.2020.00963)
Supplement: Supplementary file 1 [file Data_sheet_1.docx]

**Original Research**

***Schistosoma mansoni* FES Tyrosine kinase involvement in the mammalian schistosomiasis outcome and miracidia infection capability in *Biomphalaria glabrata***

**Short title:** SmFES roles in *Schistosoma mansoni*

Naiara Clemente Tavares1, Sandra Grossi Gava1, Gabriella Parreiras Torres1, Clara Ênia Soares de Paiva1, Bernardo Pereira Moreira1, Felipe Miguel Nery Lunkes1, Langia Colli Montresor2, Roberta Lima Caldeira1, Marina Moraes Mourão1*

1 Grupo de Helmintologia e Malacologia Médica, Instituto René Rachou, Fundação Oswaldo Cruz, Belo Horizonte, Minas Gerais, Brazil

2 Moluscário Lobato Paraense, Instituto René Rachou, Fundação Oswaldo Cruz, Belo Horizonte, Minas Gerais, Brazil

* Corresponding author:

Marina Moraes Mourão

E-mail: marina.mourao@fiocruz.br

**SUPPLEMENTARY** **FIGURES**

**
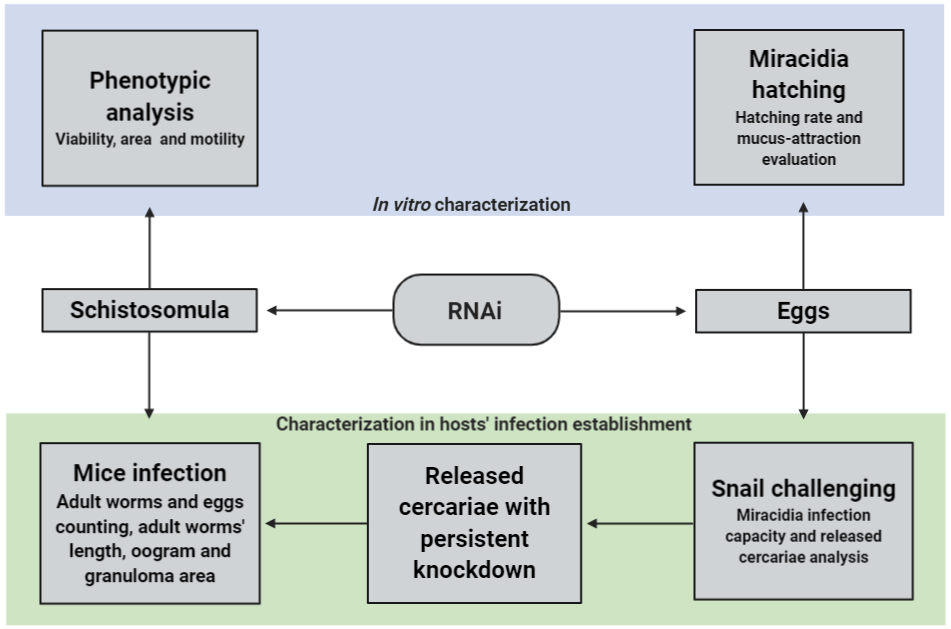
**

**Supplementary Figure S1: Simplified scheme of the methodology for SmFES characterization *in vitro* and evaluation of host infection establishment (intermediate and definitive).** Image showing the methods used to perform SmFES characterization, using RNA interference, in schistosomula and eggs/miracidia *in vitro* (blue) and mammalian and invertebrate hosts (green).

**
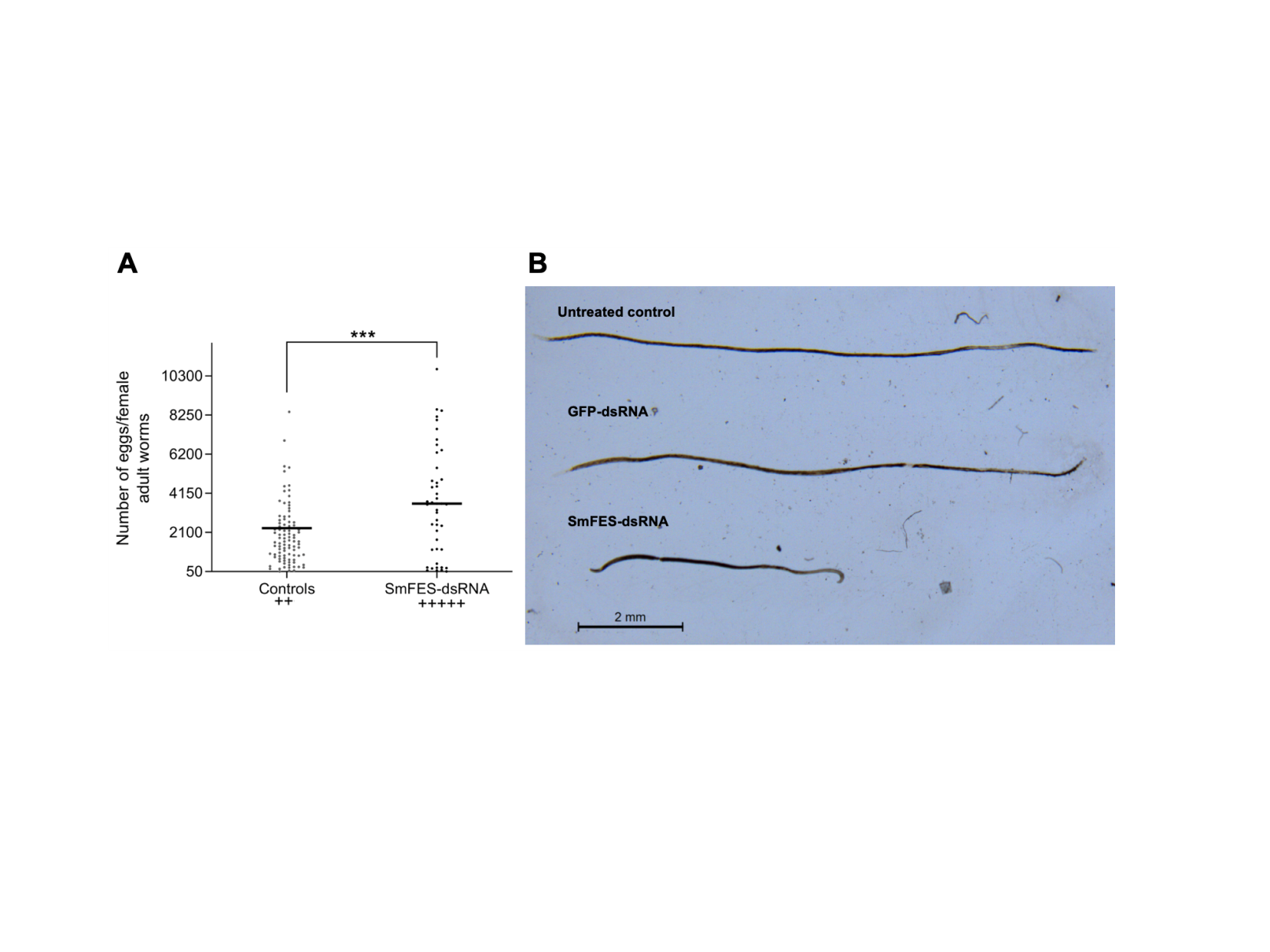
**

**Supplementary Figure S2: Fecundity and length of female adult worms recovered from mice infected with Sm*fes*- knocked-down schistosomula. (A)** Dispersion graph representing the number of total eggs per female adult worm recovered from mice after 42 days of infection with schistosomula from control groups (grey) or exposed to SmFES- dsRNA (black) for three days. Each symbol represents the fecundity of females recovered from one mouse and the cross symbols represent dead mice. Median is represented by the horizontal black line. Statistical analysis using unpaired t test with Welch's correction is represented with asterisks (***P<0.001). **(B)** Image obtained under 1.6x magnification showing females adult worms recovered from mice infected with untreated, GFP- or SmFES- dsRNA exposed schistosomula.


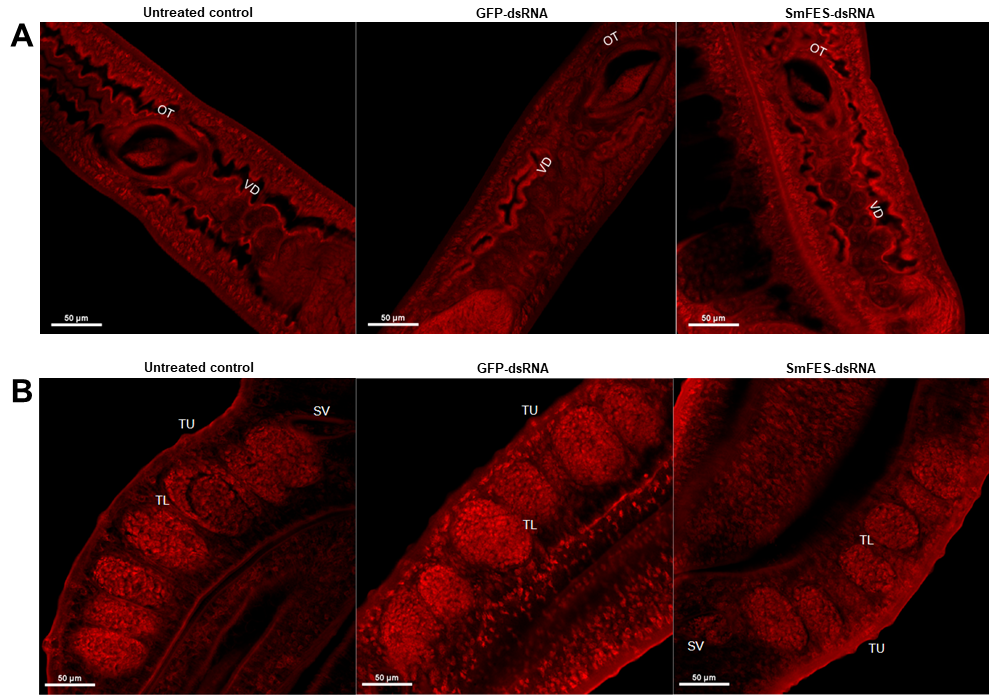


**Supplementary Figure S3: Females’ ootype and males’ testicular lobes recovered from mice infected with Sm*fes*- knocked-down schistosomula. (A)** Representative confocal images showing the ootype of females recovered from mice infected with schistosomula from untreated control, exposed to GFP- or SmFES- dsRNAs. The white bars represent the scale of 50 µm. Abbreviations: OT, ootype; VD, vitelline duct. **(B)** Representative confocal images showing the testicular lobes of males recovered from mice infected with schistosomula from untreated control, exposed to GFP- or SmFES- dsRNAs. Abbreviations: TL, testicular lobes; SV, sperm vesicle; TU, tubercle. White bars represent the scale of 50 µm.


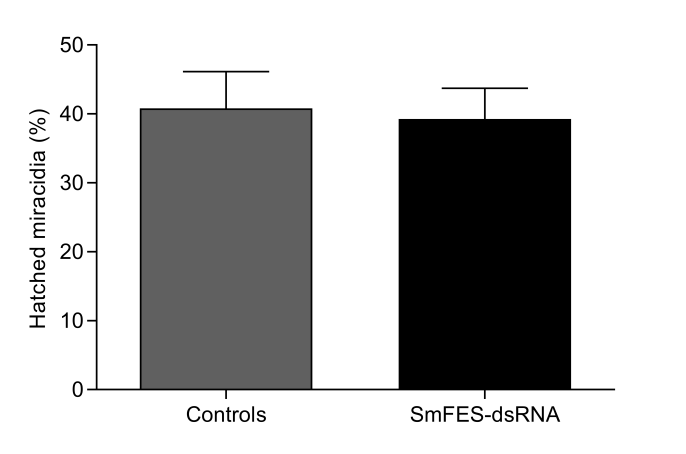


**Supplementary Figure S4: Evaluation of miracidia hatching from Sm*fes*- knocked-down eggs.** Bar graph representing the percentage of miracidia hatched from control groups (grey) or Sm*fes*- knocked-down eggs (black). Error bars are represented above the bars.

**
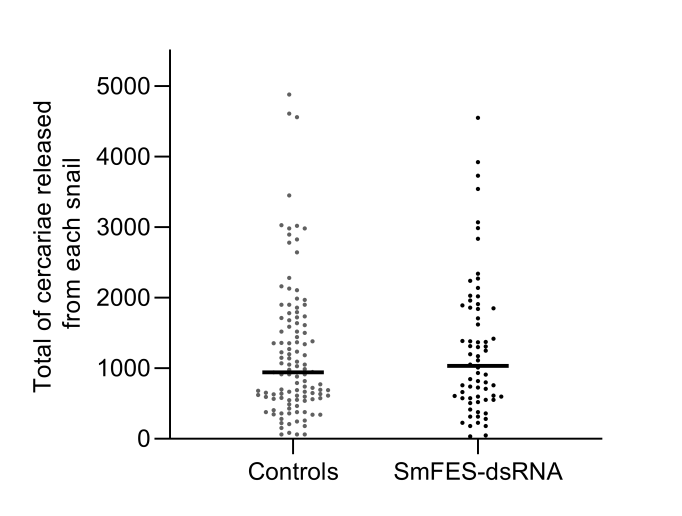
**

**Supplementary Figure S5: Assessment of number of cercariae released from *B. glabrata* infected with miraciadia derived from Sm*fes*- knocked-down eggs.** Dispersion graph representing the number of cercariae released from snails challenged with miracidia from control groups (grey) or miracidia hatched from Sm*fes*- knocked-down eggs (black). Each symbol represents the number of cercariae released from one snail. Median is represented by the horizontal black line.


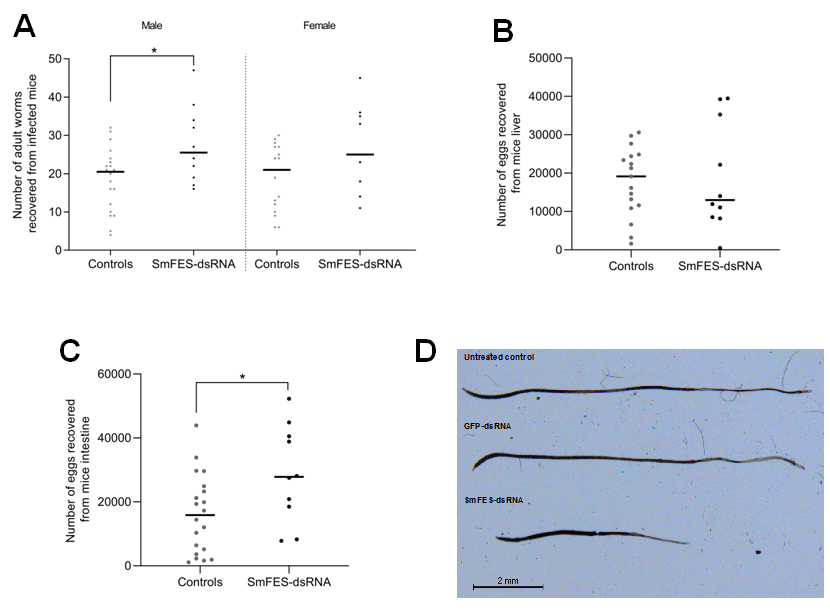


**Supplementary Figure S6: Sm*fes* knockdown effects after mammalian infection with cercariae released from *B. glabrata* infected with miraciadia derived from Sm*fes*- knocked-down eggs. Length of female adult worms recovered from mice infected with cercariae derived from Sm*fes*- knocked-down eggs.** Dispersion graph representing the number of adult worms males (left) and females (right) **(A)**, eggs from the liver **(B)** and intestine **(C)** recovered from mice after 42 days of infection with cercariae released from *B. glabrata* infected with miraciadia derived from untreated and GFP control groups (Controls-grey) or Sm*fes*- knocked-down eggs **(**black). Each dot represents the number of worms/eggs recovered from one mouse. Median is represented by the horizontal black line. Statistical analysis using Mann Whitney with is represented with asterisks (*P<0.05). **(D)** Image obtained under 1.6x magnification showing females adult worms recovered from mice infected with cercariae from control groups or Sm*fes*- knocked-down groups.

**SUPPLEMENTARY** **MOVIES**

**Supplementary Movie 1: Behavior of miracidia from untreated control group before and after the addition of *B. glabrata* mucus. (AVI)**

**Supplementary Movie 2: Behavior of miracidia from unspecific control group (GFP- dsRNA) before and after the addition of *B. glabrata* mucus. (AVI)**

**Supplementary Movie 3: Behavior of miracidia from Sm*fes*- knocked-down group before and after the addition of *B. glabrata* mucus. (AVI)**

**SUPPLEMENTARY** **TABLES**

**Supplementary Table S1: Primers sequences used for dsRNA template amplification and RT-qPCR.**

|  | Gene ID | Name | Primers sequences | Amplicon (bp) |
| --- | --- | --- | --- | --- |
| dsRNA primers | Smp_332370 | SmFES | Fwd: 5’ taatacgactcactatagggCCAACAAGCTTTGTCCGATT 3’ | 376 |
|  |  |  | Rev: 5’ taatacgactcactatagggCTGACGCGCTTGTGATTTTA 3’ |  |
|  | GFP | GFP | Fwd: 5' taatacgactcactatagggTCTTCAAGTCCGCCATG 3' | 360 |
|  |  |  | Rev: 5' taatacgactcactatagggTGCTCAGGTAGTGGTTGTC 3' |  |
| RT-qPCR primers | Smp_332370 | SmFES | Fwd: 5' TCTTTGGCATGATGATCGAG 3' | 99 |
|  |  |  | Rev: 5' GCAAGACAATTGGCAAAACA3 ' |  |
|  | Smp_900000 | COXI | Fwd: 5' TACGGTTGGTGGTGTCACAG 3' | 152 |
|  |  |  | Rev: 5' ACGGCCATCACCATACTAGC 3' |  |
